# Resistant starch diet induces change in the swine microbiome and a predominance of beneficial bacterial populations

**DOI:** 10.1186/s40168-015-0078-5

**Published:** 2015-04-16

**Authors:** Özgün C O Umu, Jeremy A Frank, Jonatan U Fangel, Marije Oostindjer, Carol Souza da Silva, Elizabeth J Bolhuis, Guido Bosch, William G T Willats, Phillip B Pope, Dzung B Diep

**Affiliations:** Department of Chemistry, Biotechnology and Food Science, Norwegian University of Life Sciences, Chr. Magnus Falsens Vei 1, P.O. Box 5003, N-1432 Ås Akershus, Norway; Department of Plant Biology and Biotechnology, University of Copenhagen, Copenhagen, DK-1871 Denmark; Adaptation Physiology Group, Wageningen University, PO Box 338, 6700 AH Wageningen, The Netherlands; Animal Nutrition Group, Wageningen University, PO Box 338, 6700 AH Wageningen, The Netherlands

**Keywords:** Growing pigs, Resistant starch, Alginate, Gut microbiota, 16S rRNA gene, Bacterial community

## Abstract

**Background:**

Dietary fibers contribute to health and physiology primarily via the fermentative actions of the host’s gut microbiome. Physicochemical properties such as solubility, fermentability, viscosity, and gel-forming ability differ among fiber types and are known to affect metabolism. However, few studies have focused on how they influence the gut microbiome and how these interactions influence host health. The aim of this study is to investigate how the gut microbiome of growing pigs responds to diets containing gel-forming alginate and fermentable resistant starch and to predict important interactions and functional changes within the microbiota.

**Results:**

Nine growing pigs (3-month-old), divided into three groups, were fed with either a control, alginate-, or resistant starch-containing diet (CON, ALG, or RS), and fecal samples were collected over a 12-week period. SSU (small subunit) rDNA amplicon sequencing data was annotated to assess the gut microbiome, whereas comprehensive microarray polymer profiling (CoMPP) of digested material was employed to evaluate feed degradation. Gut microbiome structure variation was greatest in pigs fed with resistant starch, where notable changes included the decrease in alpha diversity and increase in relative abundance of Lachnospiraceae- and *Ruminococcus*-affiliated phylotypes. Imputed function was predicted to vary significantly in pigs fed with resistant starch and to a much lesser extent with alginate; however, the key pathways involving degradation of starch and other plant polysaccharides were predicted to be unaffected. The change in relative abundance levels of basal dietary components (plant cell wall polysaccharides and proteins) over time was also consistent irrespective of diet; however, correlations between the dietary components and phylotypes varied considerably in the different diets.

**Conclusions:**

Resistant starch-containing diet exhibited the strongest structural variation compared to the alginate-containing diet. This variation gave rise to a microbiome that contains phylotypes affiliated with metabolically reputable taxonomic lineages. Despite the significant microbiome structural shifts that occurred from resistant starch-containing diet, functional redundancy is seemingly apparent with respect to the microbiome’s capacity to degrade starch and other dietary polysaccharides, one of the key stages in digestion.

**Electronic supplementary material:**

The online version of this article (doi:10.1186/s40168-015-0078-5) contains supplementary material, which is available to authorized users.

## Background

The gut microbiome of animals comprises a broad diversity of bacterial and archaeal phylotypes and is considered a separate organ due to the influence of its metabolic traits on host physiology [1]. Its key roles include modulating food intake, growth and development of the body, energy uptake from food, immune system and proliferation of epithelial cells, and resistance to infections [2]. Diet is one of the most important factors influencing gut microbiome structure and function, which indirectly modulates metabolic activities of the host [3].

Dietary fibers are defined as a large group of carbohydrates that play an important role in the gut microbiome as well as in the physiology of the host [4]. Resistant starch is an example of a dietary fiber that cannot be broken down by digestive enzymes or be absorbed in the small intestine but can be fermented by microbes in the lower gastrointestinal tract [5]. Diets rich in resistant starch have potential health benefits, such as lowering postprandial glycemia and insulinemia, enhancing absorption of minerals including calcium and iron, and prolonging the duration of satiety [6,7]. The fermentation byproducts of resistant starch (that is, short-chain fatty acids) also contribute to host health in many ways [5]. For example, butyric acid is the main energy source for colonic epithelial cells and may play a role in preventing colon cancer [8]. There are four types of resistant starch defined by their physicochemical properties, with each type affecting the gut microbiome structure differently [9]. Type 1 consists of physically inaccessible starch; type 2, granular starch; type 3, retrograded starch obtained by cooking and cooling the starch; and type 4, modified starch. Type 3 is considered the most resistant form and is totally resistant to digestive enzymes [6].

Alginate is a viscous dietary fiber consisting of guluronic acid (G) and mannuronic acid (M) that forms a gel at low pH (such as in the stomach). This gel structure slows down gastric emptying and reduces the rate of intestinal absorption of metabolizable nutrients, subsequently lowering the blood cholesterol and glucose levels [10]. Alginate may assist in the refinement of gastrointestinal barrier function and was previously shown to increase mucus layer thickness and replenishment rate, which are fundamental for the colonic mucus barrier [10]. The gel structure of alginate may also play a role in controlling obesity and type II diabetes [11] as well as limiting the adverse effects of luminal contents adsorbing a number of damaging agents such as mutagens, toxins, and carcinogens [10], thus reducing colonic exposure to these agents. Alginate-containing diets have demonstrated a satiating effect on pigs (short-term satiety) primarily due to the gel forming capability [7,12]. While fermented at a low rate by gut microbiota [10], alginate has been shown to also affect microbiome structure at some level, demonstrating its potential as a prebiotic [13,14]. However, the microbe-alginate relationship has not been evaluated in detail.

Pigs are frequently utilized as models for humans due to their similar body size, genome, digestive tract, diet type as well as other anatomical and physiological features [15,16]. Their gut microbiome also exhibits similar structural features to the extent that their use as model animals in gut microbiota studies is believed to be advantageous [17]. Previously, it has been shown that alginate and resistant starch (type 3) display different effects on the physiology and feeding patterns of growing pigs [12]. The feed intake of ALG pigs was higher than CON pigs to compensate for the reduced digestible energy intake with ALG and to result in an overall similar digestible energy intake to CON pigs. Digestible energy intake is reduced by resistant starch with increase in fermentation and more efficient use of digestible energy.

In this study, it was hypothesized that the diets containing these two contrasting dietary fibers exert different influences on the pig gut microbiome and affect important interactions and functionalities within the microbiota. Feeding trials were conducted on young animals (growing pigs) where the total energy intake should be less variable than in adult animals, as all individuals require high-energy intake for growth. Therefore, any change in microbiome structure and function in response to dietary fibers may be more visible. Microbiome analysis was conducted over a 12-week period encompassing: SSU rDNA amplicon sequencing, functional analysis of predicted metagenomes, and CoMPP analysis of plant cell wall components (PCWCs). Correlation and co-occurrence analysis were additionally conducted between the relative abundances of operational taxonomic units (OTUs) and post-digestion PCWCs.

## Results

### Feeding trials and microbiome data collection

**Figure 1:**
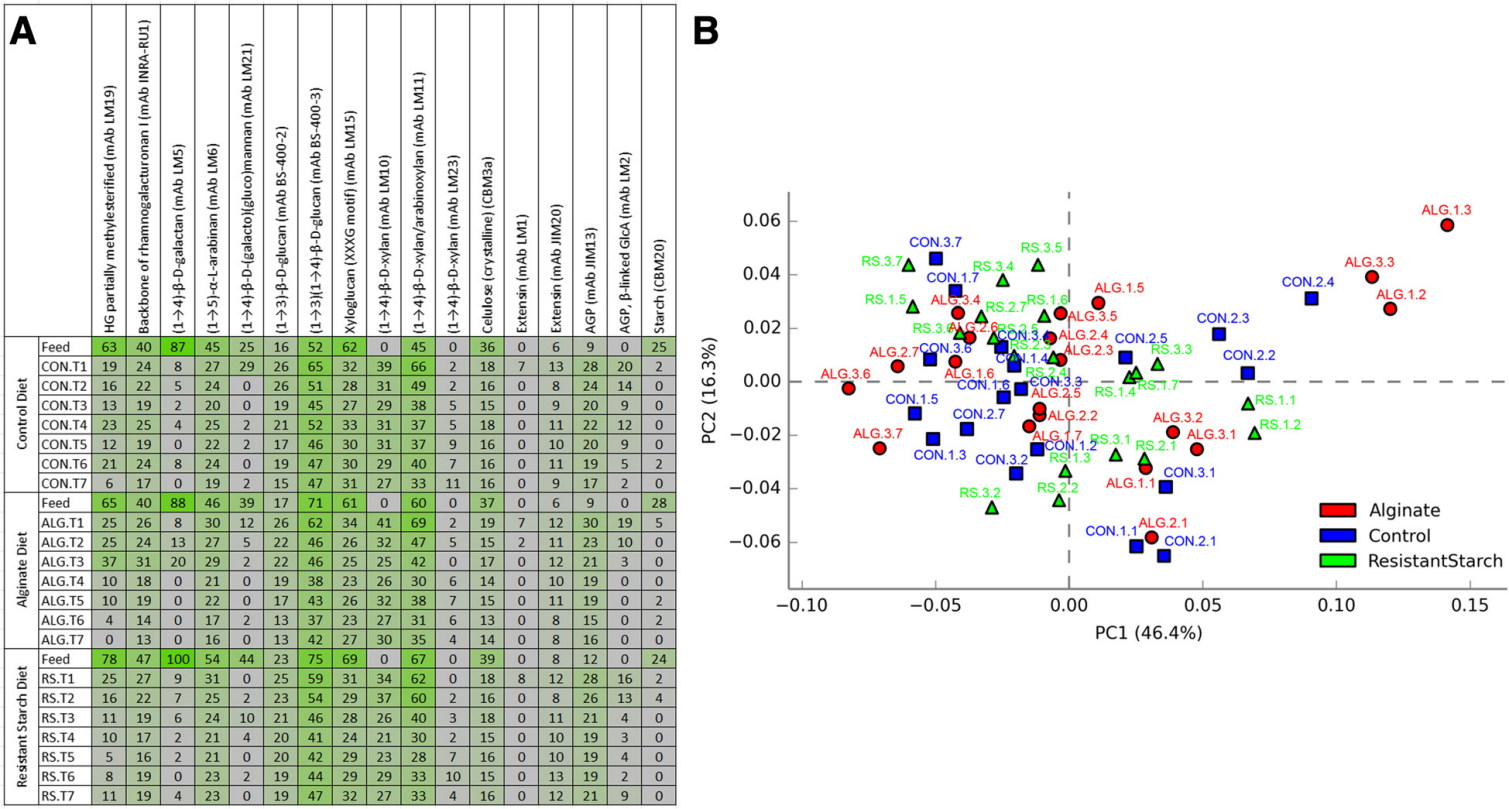
Comprehensive microarray polymer profiling (CoMPP) of plant cell wall components (PCWCs) and principle component analysis (PCA). Heatmap **(A)** shows the relative abundances of PCWCs in each sample. Color intensity is proportional to mean spot signal. T1 to T7 refer to the time-points when samples were collected. PCA plot **(B)** shows the comparison of PCWC composition between diets. Labels contain name of diet type (CON, ALG, RS), pig number for the specific diet with numbers between 1 and 3 and time-point numbers between 1 and 7 in the order (starting from T1 as first time-point). HG, homogalacturonan; AGP, arabinogalactan protein; GlcA, glucuronic acid.

In order to characterize the effects of ALG and RS on the pigs’ gut microbiomes, we assessed the community structure via 16S rRNA gene analysis. Using amplicon pyrosequencing, we obtained 251,522 SSU rRNA gene fragments in total (approximately 524 nt). Quality filtering and clustering analysis resulted in 2,621 total OTUs from 61 samples. Functional capabilities of each microbiome were predicted using KEGG pathway analyses of simulated metagenomes and compared between diet types to identify differences. CoMPP analysis was used to measure relative PCWC levels in the original feed as well as fecal samples in order to monitor changes of the individual polysaccharides and proteins that were available to the microbiome populations for ingestion (Figure 1). As expected, starch levels (detected using the CBM20 probe) were consistent in the original feed samples for all diets (Additional file 1: Table S1). No starch was detected in the fecal samples of any of the pigs, irrespective of the solubility of the starch component in their diet. Overall, a variety of pectic substrates, hemicellulosic substrates (including xyloglucans, xylans, mannans, and betaglucans), and cellulose were detected in all diet groups. The change in relative abundance of these PCWCs (decrease or increase depending on the PCWC) over time was consistent across all samples, and no differences were observed between diets (Figure 1). Alginate levels were unable to be reported via CoMPP analysis due to the lack of a suitable probe; however, previous pig feeding trials using alginate have indicated that this polysaccharide is detectable in fecal material and is not digested completely [18].

### Microbiome diversity

**Figure 2:**
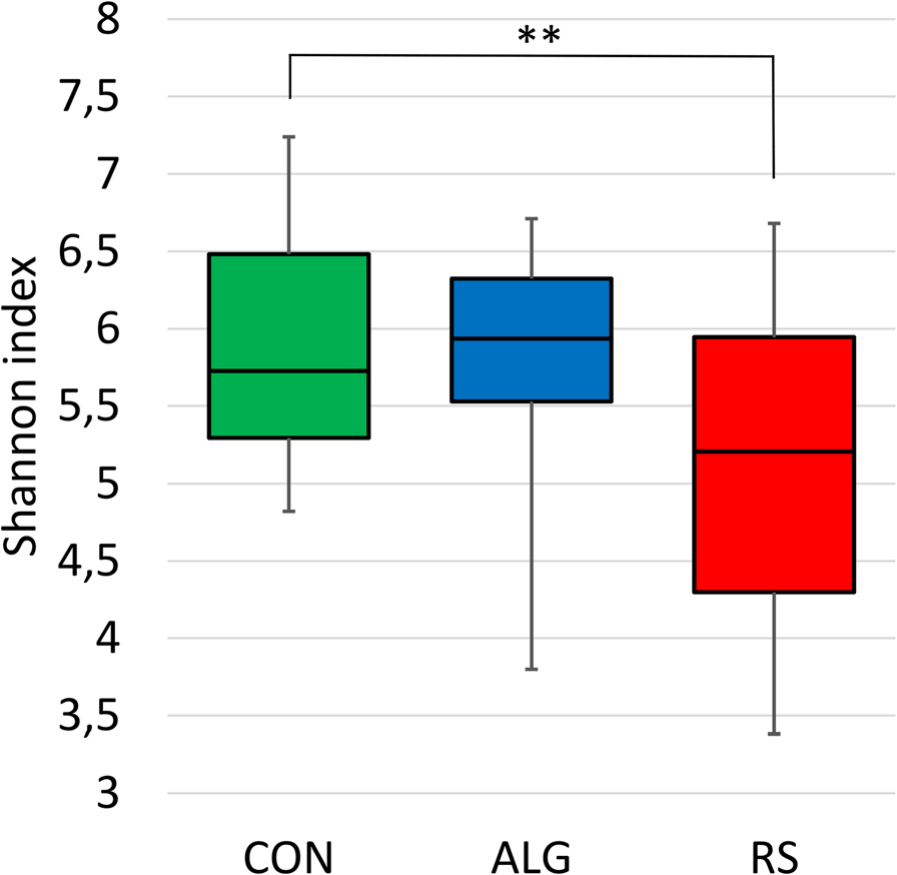
Community diversity represented by Shannon index at an OTU level for samples from each diet. Shannon indexes were calculated based on the average of ten iterations at equal subsampling size of 1,781 for each sample. Each bar represents the samples from the pigs fed with different diets; alginate-containing diet (ALG) blue, control diet (CON) green, and resistant starch-containing diet (RS) red.

Alpha diversity analyses were performed upon all samples to determine how the different diets affected the microbiome of each animal over the 12-week period. Shannon index plot (Figure 2) and rarefaction curves (Additional file 2: Figure S1) were generated for each diet group to compare the species diversity within each microbial community. Each method demonstrated that the diversity of bacterial OTUs at species level significantly (*P* < 0.01) decreased in the microbiomes of RS pigs compared to CON pigs, while there was no obvious difference in diversity between ALG and CON pigs. Moreover, time did not significantly affect bacterial alpha diversity in any of the diet groups (ANCOVA, *P* = 0.053) (Additional file 3: Figure S2).

**Figure 3:**
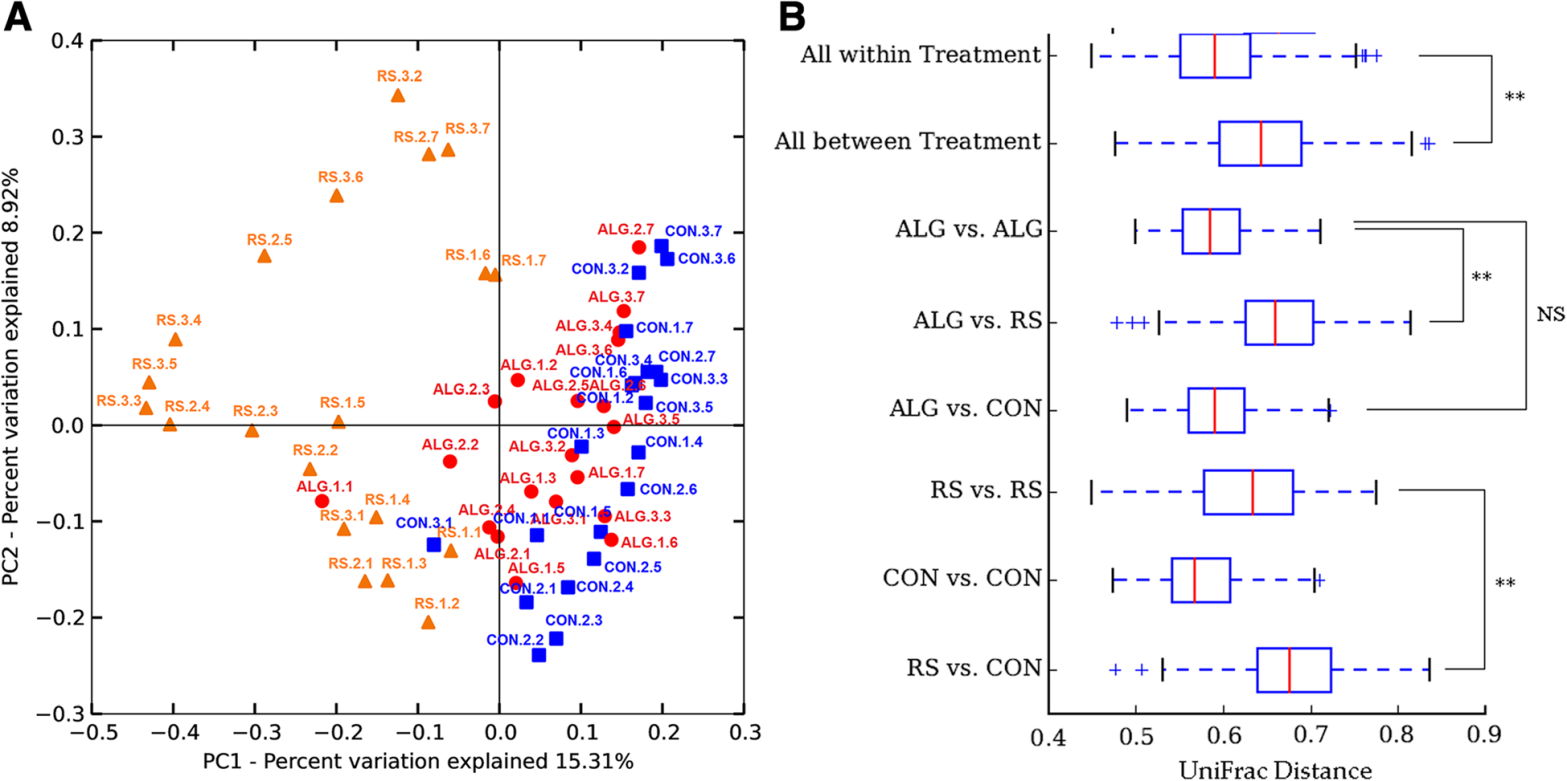
Comparison of the gut community composition. **(A)** Principle coordinate analysis (PCoA) plot generated based on the calculated distances in an unweighted UniFrac matrix. Samples were grouped by color and shape in terms of diet group they belong to; alginate-containing diet (ALG) red (circle), control diet (CON) blue (square), and resistant starch-containing diet (RS) orange (triangle). Labels contain name of diet type (CON, ALG, RS), pig number for the specific diet with numbers between 1 and 3, and time-point numbers between 1 and 7 in the order (starting from T1 as first time-point). **(B)** The statistical significances of differences in unweighted UniFrac distances between diets. Significance degree (calculated using Student’s *t*-test with 1,000 Monte Carlo simulations) is represented as no significance (*P* > 0.05) with NS; *P* < 0.05 with one star (*); *P* < 0.005 with two stars (**).

To explicitly compare the microbiomes of the individual animals used in this study, distance matrices were calculated by unweighted UniFrac [19], visualized via principle coordinate analysis (PCoA) (Figure 3A), and statistical analyses were performed on distance matrices for significance testing (Figure 3B). Pigs fed with the same diet tended to cluster together (Figure 3A), while time did not significantly affect the bacterial community composition of fecal samples within each diet over the 12-week period (Additional file 4: Table S2). CON pigs were shown to cluster in close proximity after samples T2 to T3, indicating that they were acclimatized to their diet within 1 to 3 days after the start of the prebiotic diet. The relatively sporadic clustering between the three pigs fed with the same diet and sampled at the same time was possibly due to the inter-individual variation (Additional file 5: Figures S3 and Additional file 4: Table S2), a commonly observed phenomenon [20,21]. As expected, the first time-point (T1, day −7) samples of all diets had similar microbiome structure since all pigs were fed with the same commercial basal diet at this time. However, from time-point 2 (T2, day 1) when pigs were fed with different diets, their microbiomes started to diverge from each other, with those from RS pigs in one direction while those from ALG and CON pigs jointly in another direction. The structural shift of the microbiome of RS pigs compared to CON and ALG pigs were statistically significant, whereas ALG pigs had similar microbiome composition to CON pigs (Figure 3B).

### Taxonomic affiliations

Overall, the microbiomes of the individual pigs were dominated by the phyla Firmicutes (88.2% in CON pigs, 90.1% in ALG pigs, and 88.3% in RS pigs) and Bacteroidetes (9.7% in CON pigs, 8.6% in ALG pigs, and 10.2% in RS pigs). The other phyla present in low abundance (less than 2.1%) were Actinobacteria, Cyanobacteria, Spirochaetes, TM7 (candidate division), Tenericutes, and a number of unclassified bacteria. Although most of these phyla were present in samples across all diets, Spirochaetes were not detected in RS pigs and TM7 was observed only in ALG pigs.

**Figure 4:**
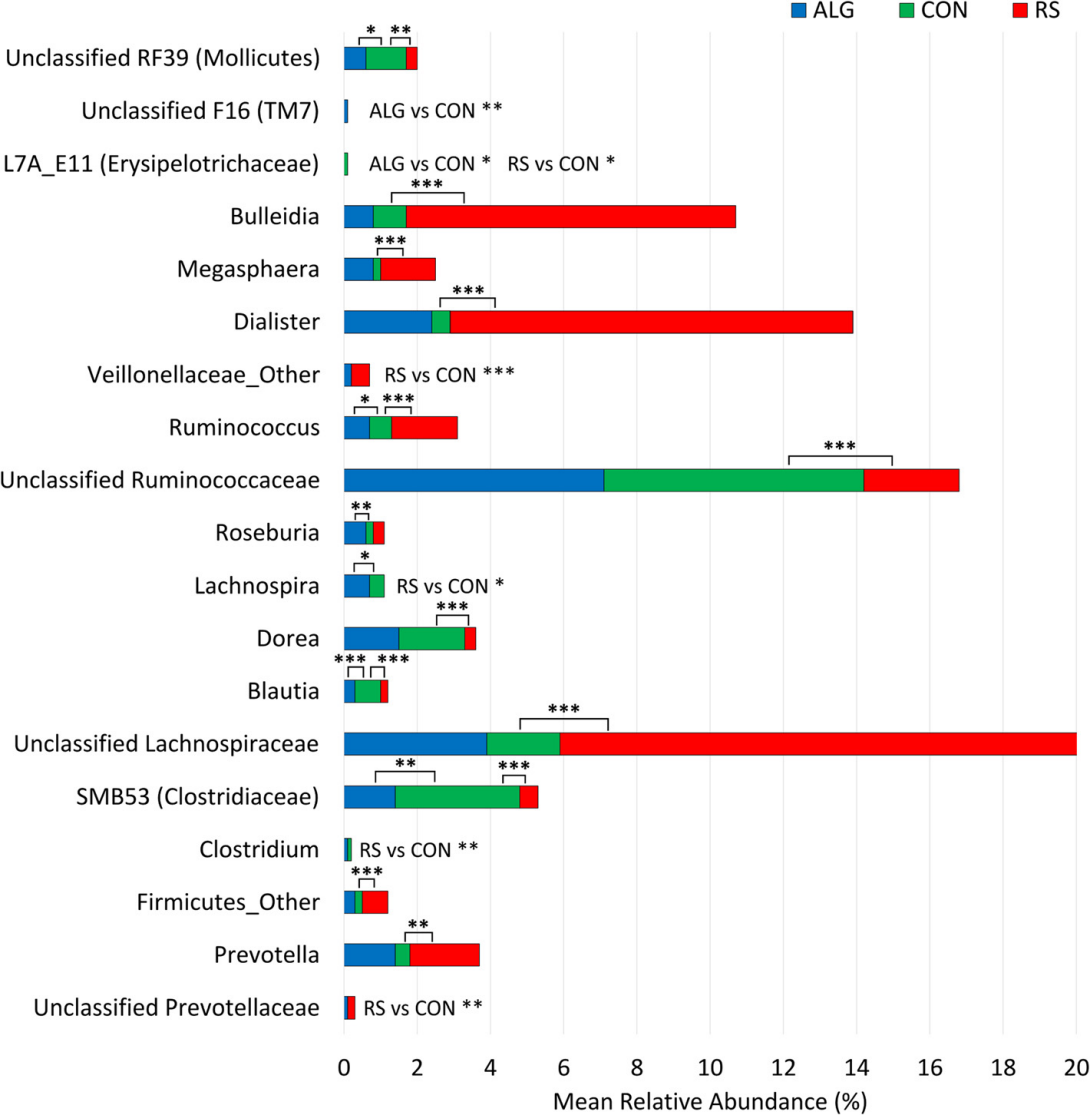
Significantly different bacterial genera in relative abundance between different diets. Genera that have different relative abundances in ALG or RS pigs compared to CON pigs were determined by ANCOVA. The shown mean relative abundance percentages of the taxa were calculated using all samples taken over time within each diet. Significance degree is represented with stars; *P* < 0.05 with one star (*); *P* < 0.01 with two stars (**); *P* < 0.001 with three stars (***). The significance was stated next to the bar together with the abbreviations of compared diets (ALG, CON, and RS) when the bar does not appear for at least one of the diets due to a very low relative abundance percentage.

At deeper taxonomic levels, a greater number of significant differences were observed (Additional file 6: Figure S4). At the family level, the following families were more abundant in RS pigs than CON pigs: Erysipelotrichaceae (*P* < 0.001), Veillonellaceae (*P* < 0.001), Lachnospiraceae (*P* < 0.01), an undefined Firmicutes family (*P* < 0.001), and Prevotellaceae (*P* < 0.001). In contrast, the families unclassified RF39 (affiliated to Mollicutes) (*P* < 0.01) and Clostridiaceae (*P* < 0.001) appeared significantly less abundant in RS pigs than in CON pigs. The relative abundance of only unclassified F16 family (affiliated to TM7) (*P* < 0.01) was higher in the microbiome of ALG pigs than that of CON pigs, whereas the unclassified RF39 (affiliated to Mollicutes) (*P* < 0.05) and Clostridiaceae (*P* < 0.001) were less abundant. At the genus level, ANCOVA resulted with many genera with significant relative abundance differences in RS and less in ALG compared to CON (Figure 4). *Bulleidia* (*P* < 0.001), *Megasphaera* (*P* < 0.001), *Dialister* (*P* < 0.001), an undefined Veillonellaceae genus (*P* < 0.001), *Ruminococcus* (*P* < 0.001), unclassified Lachnospiraceae genus (*P* < 0.001), an undefined Firmicutes genus (*P* < 0.001), *Prevotella* (*P* < 0.01), and unclassified Prevotellaceae genus (*P* < 0.01) were more abundant in RS pigs compared to CON pigs, while unclassified RF39 genus (affiliated to Mollicutes) (*p* < 0.01), L7A_E11 (affiliated to Erysipelotrichaceae) (*p* < 0.05), Unclassified Ruminococcaceae (*P* < 0.001), *Lachnospira* (*P* < 0.05), *Dorea* (*P* < 0.001), *Blautia* (*P* < 0.001), SMB53 genus (affiliated to Clostridiaceae) (*P* < 0.001), and *Clostridium* (*P* < 0.01) had a significantly lower relative abundance. In the ALG pigs, the most notable observation was the significantly higher relative abundance of unclassified F16 genus (affiliated to TM7) (*P* < 0.01), *Ruminococcus* (*P* < 0.05), *Roseburia* (*P* < 0.01), and *Lachnospira* (*P* < 0.05) compared to CON pigs.

**Figure 5:**
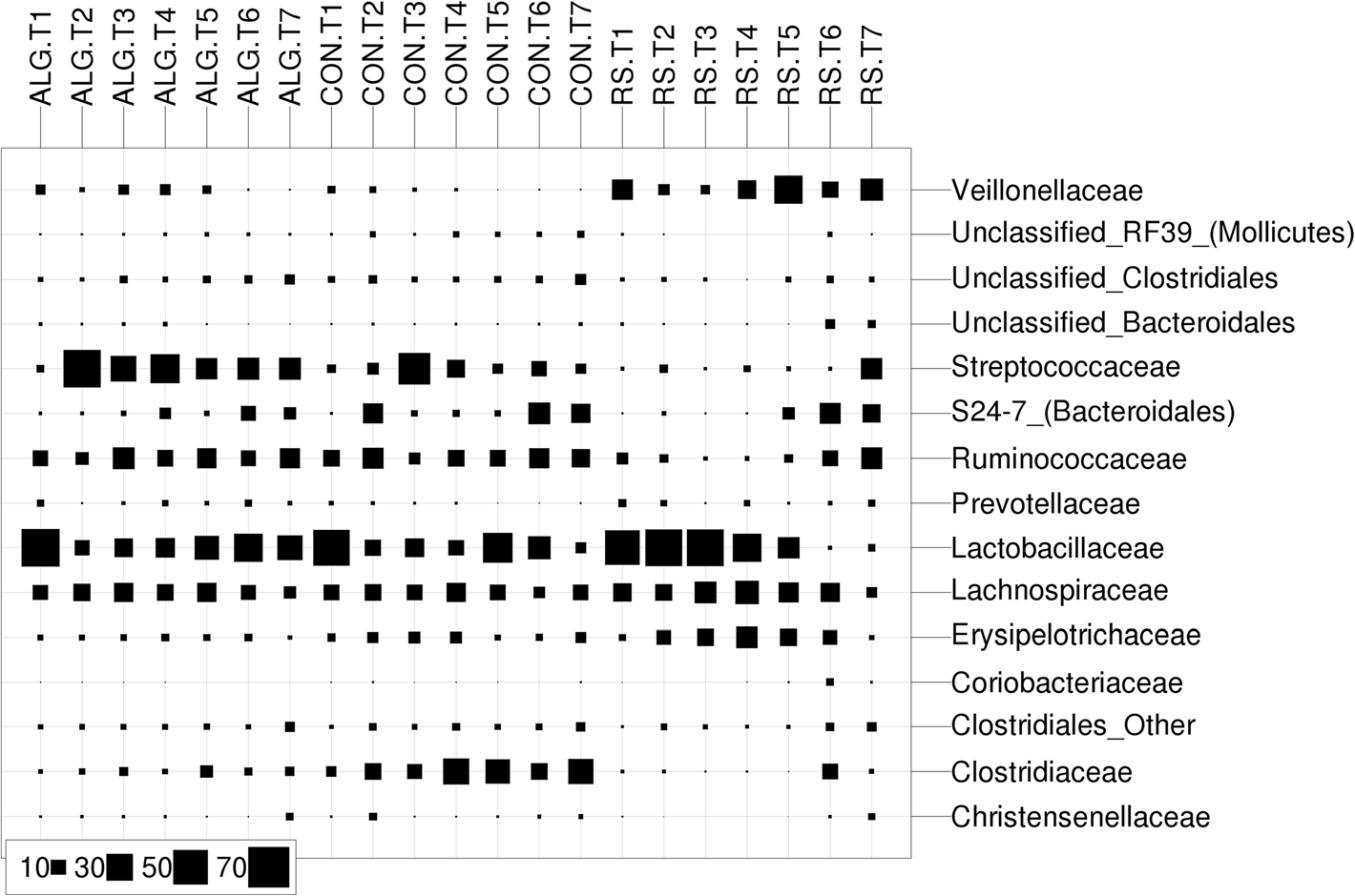
The relative abundances of bacterial families for each fecal sample over time. The size of each square represents the mean relative abundance of bacterial families (%) for the indicated time-point and was determined from fecal samples of three pigs that were fed with the same diet. Samples were ordered in terms of time within each diet and labeled beginning with diet type (ALG, CON, and RS) and time-point from T1 to T7 (T1: day −7, T2: day 1, T3: day 3, T4: day 7, T5: week 3, T6: week 7 and T7: week 12).

The relative abundances of some of the bacterial families within dietary groups tended to show variations over time (Figure 5). Streptococcaceae and Lactobacillaceae showed an opposing trend in relative abundance variation over time in all diets. Moreover, relative abundance of some families including Lachnospiraceae, Erysipelotrichaceae, and Veillonellaceae varied over time (becoming more abundant and less abundant over time) in an opposing manner to some other families such as Ruminococcaceae, S24-7, Clostridiaceae, unclassified Clostridiales, and unclassified Bacteroidales, particularly in RS pigs (Figure 5). The patterns of these contrasting changes between particular families were supported by Pearson’s correlations, which were consistent with the different diet types (Additional file 7: Figure S5). For example, specific families that positively correlated with one another and to the RS diet were often negatively correlated to other groups that were positively correlating to the CON diet. ALG correlated positively with Streptococcaceae only, while RS correlated positively with many families such as Veillonellaceae, Lachnospiraceae, Erysipelotrichaceae, and Prevotellaceae that became predominant by RS.

### Imputed microbiome function

**Figure 6:**
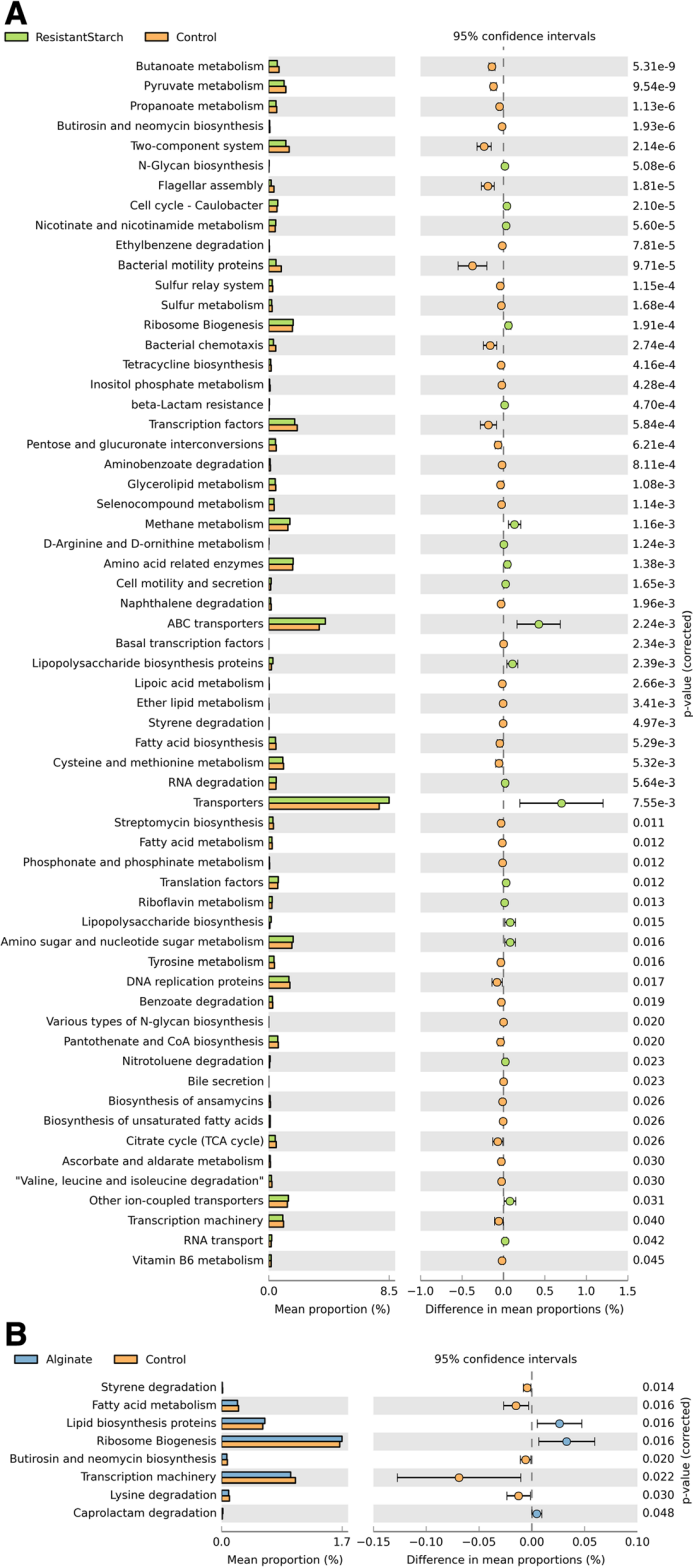
Imputed metagenomic differences between ALG and RS pigs compared to CON pigs. The relative abundance of metabolic pathways encoded in each imputed sample metagenome was analyzed using STAMP [63]. Extended error bars show significantly different KEGG pathway maps in RS **(A)** and ALG **(B)** pigs compared to CON pigs (*P* < 0.05, confidence intervals = 95%).

Given the structural changes within the microbiome of RS and ALG pigs compared to CON pigs, we subsequently examined whether the contrasting diets would also cause functional changes within each microbiome. In the absence of shotgun metagenomic sequencing data, we applied PICRUSt [22] to our 16S rRNA gene survey to predict metagenome functional content. PICRUSt is a computational approach in which evolutionary modeling is used to predict the present gene families from 16S data and a reference genome database [22]. The imputed relative abundances of KEGG pathways in each respective sample were used to predict changes in metabolic function within the microbiomes of ALG and RS pigs compared to CON pigs (Figure 6). The RS diet was predicted to significantly affect (*P* < 0.05) a greater number of KEGG pathways (sevenfold) in the gut microbiome, whereas the ALG diet seemingly had a reduced impact on microbiome function compared to CON diet. The KEGG pathways that exhibited the greatest statistical difference in RS and CON pigs were butanoate, pyruvate, and propanoate metabolism, with all having a higher predicted relative abundance in CON pigs. Interestingly, there were no significant differences in the starch and sucrose metabolism KEGG pathway between RS pigs and ALG pigs compared to CON pigs although a significant difference was observed at one time-point (T3) (*P* = 0.046) between RS and CON pigs (Additional file 8: Figure S6). While this KEGG pathway map encompasses starch conversion, it also includes cellulose, xylan, betaglucan, and pectin conversion (http://www.genome.jp/kegg/kegg2.html, map00500), which are all key PCWCs that were detected using CoMPP analysis.

### OTU-PCWC correlations

**Figure 7:**
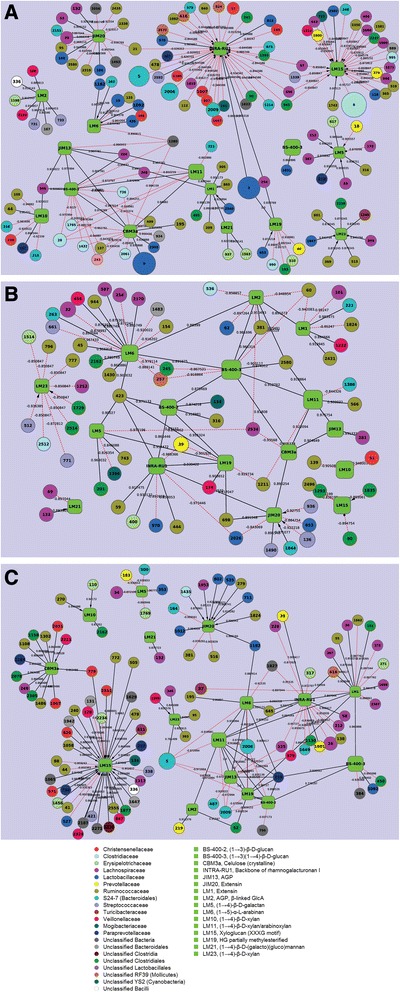
Correlation networks of OTUs and PCWCs in each diet. OTUs were grouped at 97% SSU rRNA gene identity and the networks were plotted based on eLSA with significant local similarity scores (*p* < 0.001). **(A)**, **(B)**, and **(C)** networks represent CON, ALG, and RS, respectively. The numbers on nodes are OTU numbers, and PCWCs are labeled with their targeting monoclonal antibodies. All PCWCs are shown by one color (green) while OTUs belonging to different families are represented by different colors (see legend). The size of each node is proportional to the value of relative abundances. Solid edges (black) are positively associated while dashed edges (red) are negatively associated. Edges without any tip show co-occurrence without time delay; while one, two, and three time-point delays are indicated on the affected feature with an arrow, circle, or diamond tip, respectively. HG, homogalacturonan; AGP, arabinogalactan protein; GlcA, glucuronic acid.

To investigate correlation/co-occurrence of PCWCs and bacterial taxa, extended local similarity-based networks were applied as they can be used to evaluate correlations between two data types over time. Many different OTUs that were affiliated to various families co-occurred or correlated significantly (*P* < 0.001) with PCWCs in different diet pigs. Although the relative levels of PCWCs did not show any difference between diets (Figure 1), the number of the OTUs varied in the CON, ALG, and RS networks (Figure 7 and Additional file 9: Figure S7). OTUs affiliated to the Ruminococcaceae, Lachnospiraceae, and Lactobacillaceae families were the most abundant taxa that co-occurred/correlated with the PCWCs in all diets.

In the CON network, rhamnogalacturonan I (INTRA-RU1) and xyloglucan (LM15) exhibited the highest number of correlations with different OTUs. These polysaccharides typically had negative correlations (with a one time-point delay such that the shift on OTU relative abundance affects polysaccharide relative abundance with a delay of one time period), suggesting that an increase in the relative abundance of these OTUs was correlated to a decrease in the relative levels of these polysaccharides in CON pigs. Within the RS network, many taxa co-occurred with xyloglucan (LM15), although the OTUs were affiliated to different lineages and the majority of correlations were positive with one time-point delay. Most of the highly abundant S24-7 OTUs (OTU5, OTU2006, and OTU2009) negatively co-occurred in the RS network with more than one PCWC, including arabinan (LM6), arabinogalactan protein (AGP: JIM13), homogalacturonan (HG: LM19), β-(1,3) glucan (BS-400-2), and xylan/arabinoxylan (LM11). However, this varied in the CON network as the same OTUs were only negatively correlated with rhamnogalacturonan 1 (INTRA-RU1). The number of OTUs that exhibited correlations in the ALG network was relatively lower (almost half of CON and RS networks), with the most prominent being negative correlations (with a one time-point delay) between the unclassified Clostridiales and xylan (LM23) and xyloglucan (LM15) as well as Streptococcaceae and xylan (LM23) and glycoproteins (extension: JIM20).

## Discussion

SSU rRNA gene amplicon sequence analysis and CoMPP of PCWCs were used to evaluate the effects of dietary fibers (alginate and type 3 resistant starch) on the gut microbiome of growing pigs during a 12-week feeding experiment. The fibers assessed in this study have contrasting properties, the most prominent being the gel-forming capacity of alginate fibers whereas resistant starch is resistant to the host’s digestive enzymes but fermentable by gut flora in the lower intestine. Findings by Souza da Silva *et al.* [12,23] demonstrated that these two fibers affected feeding patterns and physiology of growing pigs in different ways. The feeding patterns were affected less by alginate addition in the diet compared to resistant starch addition in a manner that only cumulative and average daily feed intake increased in ALG pigs compared to CON pigs, to achieve similar digestible energy intake. Moreover, both diets increased the relative empty weight of the colon, but only RS increased the weight of the total gastrointestinal tract. This is conceivably the result of an increase in bacterial mass and fermentation end-products [24] or an increase in metabolically active tissue in the colon [12,25]. The gut microbiota plays an important role in host physiology [26], and a different impact on community composition resulting from ingestion of these dietary fibers is therefore expected to occur due to their different physicochemical and metabolic properties. This study showed that resistant starch (type 3) had significant effect on gut community structure of growing pigs while the community composition in ALG pigs was similar to that in CON pigs. Moreover, the demonstrated shift in microbiome structure of RS pigs was specific to diet type in spite of the inter-individual variations.

Alpha diversity within the microbiome was lower in RS pigs compared to CON pigs, which is most likely due to the selection of particular genera among the Firmicutes. Many bacterial lineages exhibited shifts in relative abundances after the commencement of the different diets, with RS pigs being the most pronounced. In some previous studies, performed with varied methods and models, it has been shown that type 2 resistant starch increases *Ruminococcus bromi* and *Eubacterium rectale*, while type 4 resistant starch promotes the growth of *Bifidobacterium adolescentis* and *Parabacteroides distasonis* in human subjects [27], and that *Bifidobacterium*, *Akkermansia*, and *Allobaculum* are increased by type 2 resistant starch in mouse models [28]. Similarly, type 3 resistant starch has led to the increased relative abundances of *E. rectale*, *Roseburia* spp., and *R. bromii* in mouse models [29], *E. rectale*, *Roseburia*, Clos IV *Ruminococci* and *Oscillospira* in obese male humans [30], and *R.bromii* in colonic samples of pig models [31]. In the present study, we observed an insignificant increase in *Roseburia* relative abundance in the microbiome of RS pigs, whereas *Eubacterium* was not detected in any of the pigs irrespective of diet. The *Ruminococcus* genus, including *R. bromii,* which is known for its ability to degrade resistant starch [32], had a significant increase in RS pigs. In addition, a broad diversity of bacterial genera increased in relative abundance due to RS, including *Bulleidia, Megasphaera, Dialister,* an unclassified Lachnospiraceae genus, and *Prevotella.* The increase in some of these bacterial lineages was also observed previously in growing pigs after 14 days of feeding with type 3 RS compared to CON pigs [23,33]. The increase in relative abundances of *Ruminococcus* (threefold) and *Prevotella* (nearly fivefold) in RS pigs compared to CON pigs is notable due to their ability to use polysaccharides to produce short-chain fatty acids [34] that are known to play a protective role against gut inflammation [35] and be used as an energy source for the host [6]. The predominance of Lachnospiraceae in RS pigs is also noteworthy as previous mouse studies [36] have demonstrated that their presence can lead to a reduction in *Clostridium difficile* colonization, which is an important pathogen for pigs and humans [37-39]. We found an increase in Lachnospiraceae as well as a decrease in Clostridiaceae in RS pig. Although there was no direct correlation between these families, the interaction between specific species affiliated to these families need to be investigated further. *Bifidobacterium,* which is known for its minority in pig intestine [40,41], was not detected in any of pigs regardless of diet type.

Compared to resistant starch, alginate has a low fermentability [10], however, it has been demonstrated to have a positive impact on the total bacterial count in the human fecal microbiome *in vitro* and is believed to have prebiotic effects [13,14]. Its consumption has been shown to result in a significant increase in the number of *Bifidobacteria* and a decrease in the number of Enterobacteriaceae in healthy human subjects [42], whereas the relative abundance of *Bacteroides capillosus* has also been demonstrated in the cecum of rats fed with sodium alginate [43]. In ALG pigs, less variation within the microbiome structure than RS pigs was observed when they both were compared to CON pigs. However, we observed that alginate affects the gut bacterial community via altering the relative abundances of some families and genera. In particular, Clostridiaceae-affiliated phylotypes experienced decreased relative abundance in ALG pigs similar to the RS pigs when compared to the CON animals.

Time did not have a significant influence on alpha and beta diversity metrics within any of the diets. This can be explained by the short experimental period and the maturity (3 to 6 months old) of these growing pigs, which were principally in a child-to-early-adolescent life stage. Diversity levels during this period are typically more comparable to adults and generally more stable than those during the infant period [44-46]. The natural age of completion of weaning in pigs differs from 9 to 20 weeks [47], whereas the onset of puberty in pigs can be as early as 5 months in female pigs [48]. The 3-month-old pigs used in this study were weaned before the commencement of the feeding trials and had only a few months to puberty. Despite the relative stability of diversity metrics, the relative abundances of some families did change over time. These alternating variations between families that were correlated negatively with each other (Additional file 7: Figure S5) may indicate the competitive interactions within the community as a result of substrate change in the community with addition of fibers.

The shifts in microbiome structure of ALG and RS pigs were consistent with imputed functional predictions. ALG had little effect on predicted microbiome function, which was expected since there was little change in the microbiome structure. In contrast, RS pigs experienced greater microbiome structural shifts, subsequently resulting in more predicted changes in the relative abundance of imputed KEGG pathway maps. Many of the significantly altered imputed functions in RS pigs were related to fatty acid metabolism such as butanoate and propanoate. Resistant starch is known to play an important role in fatty acid production in the gut [49,50], therefore it was surprising that imputed butanoate and propanoate metabolisms were associated negatively with RS compared to the CON diet. The KEGG starch and sucrose metabolism pathway map which contains the majority of reactions involving starch, cellulose, xylan, and pectin degradation was not significantly influenced by RS or ALG with all time-points considered. Assessing the individual samples taken over the 12-week time period revealed a similar pattern with the exception of one sample (T3), which demonstrated a higher imputed representation of this KEGG pathway in RS pigs. This result seems to correspond well with CoMPP analysis of PCWCs, which showed polysaccharide degradation consistency between diets over time.

RS and ALG diets were found to influence OTU and PCWC correlations/co-occurrences over time, with the same PCWCs in CON, ALG, and RS pigs often correlated with different OTUs. This was expected given that alginate and resistant starch caused varying changes to microbiome structure, whereas the PCWC availability in the microbiome is believed to be largely unchanged. This was clearly illustrated for the hemicellulose polysaccharide xyloglucan (target of probe LM15), for which the total number and OTU affiliation of correlations varied substantially between CON, ALG, and RS pigs (Figure 7). Many OTUs affiliated to the Ruminococcaceae and Lachnospiraceae families were positively correlated to PCWCs and thus inferred in PCWC metabolism in growing pigs regardless of diet type. Both of these families are well known for degradation of complex plant material (for example, cellulose, hemicellulose) in the mammalian gut environment [51].

## Conclusions

In conclusion, RS exhibited the strongest structural variation compared to ALG, which is likely resultant from the contrasting physicochemical properties of these dietary fibers. The increase in relative abundance of Lachnospiraceae-, *Prevotella*- and *Ruminococcus*-affiliated phylotypes in RS pigs can be considered as desirable traits given the reputation of these groups in fiber degradation and production of short chain fatty acids. Moreover, resistant starch and to a lesser extent alginate, influenced the imputed functionality of predicted metagenomes and correlation between bacterial phylotypes and PCWCs. With all data collectively considered, we speculate that despite the microbiome structural differences between diets, functional redundancy exists in the key metabolic stage of polysaccharide degradation. The observed stability in the imputed KEGG starch and sucrose metabolism pathway and consistent PCWC availability between diets supports this hypothesis. Furthermore, the variation in OTU-PCWC correlations between the different diets suggests that different phylotypes possibly drive PCWC utilization within each feeding regime. These hypotheses require further detailed metagenomic investigations to deduce the metabolic capabilities of key uncultured populations within the microbiome of pigs, and form the basis of our ongoing efforts.

## Methods

### Study design and sampling

Nine pigs (approximately 3 months old) selected for this study were housed, fed, and sampled at the Nutreco Swine Research Centre facilities, Sint Anthonis, The Netherlands [12]. Each group of three pigs was fed with one of three diets: control (CON) containing no prebiotic dietary fiber, alginate-containing (ALG) and retrograded (Type 3) resistant starch-containing (RS). The control diet was formulated to contain 40% digestible starch, and other diets were formulated from control diet by exchanging alginate (sodium alginate in dry form) or resistant starch (retrograded tapioca starch) for digestible starch on a dry matter. (Additional file 1: Table S1, for further diet details refer to [12]). Weight measurements were also performed during the feeding period. There was no significant difference between the weights of the pigs fed with different diets, although all pigs achieved a final weight (99.4 ± 6.7 kg) greater than three times of the initial weight in the experiment (31.7 ± 1.4 kg). The pigs were labeled with respect to diet they were fed with, such as CON.1, CON.2, CON.3, ALG.1, ALG.2, ALG.3, RS.1, RS.2, and RS.3. All pigs originated from the same batch consisting of castrated males with the exception of one female (ALG.2) and were unrelated except for two siblings (ALG.1 and RS.3). Each pig was fed with the aforementioned diet over a 12-week period (T2 to T7), and fecal samples were collected at seven different time-points (T1: day −7; T2: day 1; T3: day 3; T4: day 7; T5: week 3; T6: week 7; T7: week 12). All pigs were fed with a commercial basal diet for 3 weeks before the experiment commenced and the first fecal sample collection (T1). The adaptation to the diets was performed by gradual exchanging of the commercial diet for one of the CON, ALG, and RS during a 7-day period before T2, from which point the complete differentiation in diets started. The 7-day transition period entailed the following stages: 2 days of the animals being fed with the commercial diet (100%); the third day, the commercial diet was supplemented with 20% of the different prebiotic diets; and from days 4 to 7, the percentage of the prebiotic diet was increased in 20% increments until the prebiotic diet reached 100% (T2). A total of 61 fecal samples were used because the rectum of two pigs were empty at the time of collection of fresh fecal samples (pig ALG.1 at T4 and pig RS.3 at T6), and these two samples were subsequently not available. Fresh fecal samples were homogenized and kept at −20°C until analysis.

### Cell dissociation and DNA extraction

Bacterial cells were harvested from 0.3 *g* of frozen feces using a cell dissociation protocol as described previously [52]. The samples were suspended in acidic dissociation buffer [53] containing (v/v) 0.1% Tween 80, 1% methanol, and 1% tert-butanol, and cells were harvested from supernatant by quick centrifugation. These steps were repeated five times to increase cell yield. Cell pellets were collected by high-speed centrifugation (14,500 *g* for 5 min) and washed with a wash buffer containing 10 mM TrisHCl and 1 M NaCl. DNA extraction was performed as described in [54] with small modifications. The cells were re-suspended in RBB + C lysis buffer containing 500 mM NaCl, 50 mM TrisHCl, and 50 mM ethylene diamine tetraacetic acid (EDTA) and incubated with lysozyme and mutanolysin enzymes at 37°C for 30 min. Further lysis was carried out by addition of 4% sodium dodecyl sulfate (SDS) and incubation at 70°C for 20 min, mixing the tube by inversion every 5 min. Cetyltrimethyl ammonium bromide (CTAB) buffer was used for DNA precipitation. After repeated treatments with chloroform and phenol/chloroform/isoamyl alcohol, DNA was precipitated by isopropanol, washed once with ethanol, re-suspended in water, and kept at −20°C until further analysis.

### Bacterial SSU rRNA gene amplification and 454 pyrosequencing

The SSU rRNA gene fragment hyper variable regions V1 to V3 were amplified from extracted DNA using 8F-515R bacteria-specific primers. The forward primer is a combination of the 454 fusion adapter B sequence and universal bacterial primer 8F, 5′-*CCT ATC CCC TGT GTG CCT TGG CAG TCT CAG CAA CAG CT*A GAG TTT GAT CCT GG-3′. The reverse primer is a combination of the 454 fusion adapter A sequence including a unique 8 nt multiplex barcode, represented by Ns, and universal bacterial primer 515 R, 5′-*CCA TCT CAT CCC TGC GTG TCT CCG ACT CAG NNN NNN NN*T TAC CGC GGC TGC T-3′. Each PCR reaction consisted of 25 μl iProof High-Fidelity Master Mix (BioRad, Hercules, CA, USA), 0.2 mM forward primer, 0.2 mM reverse primer, 400 ng template DNA, and sterile water to a total volume of 50 uL. The following PCR program was used: denaturation at 98°C for 30 s, 30 cycles of 10 s at 98°C, 30 s at 58°C, and 40 s at 72°C and a final extension at 72°C for 7 min. PCR product concentrations were measured by Qubit® fluorometer using Qubit® dsDNA BR Assay Kit (Invitrogen, Eugene, OR, USA) and checked by gel electrophoresis (1% agarose gel). All PCR products were pooled into one tube in equal amounts and run on a 1% agarose gel. The band containing pooled PCR products was excised and purified using NucleoSpin Extract II kit (Macherey-Nagel, Düren, Germany). Pyrosequencing was performed on the 454 GS FLX sequencer (Roche) at the Norwegian Sequencing Center (Oslo, Norway).

### Analysis of 16S rRNA gene sequences

The sequencing reads were processed and analyzed using Quantitative Insights Into Microbial Ecology (QIIME) version 1.7.0 [55]. Reads of quality lower than 25, lacking a barcode, and/or shorter than 400 or longer than 600 nt were not analyzed further. The remaining reads (93%) were multiplexed to samples based on their nucleotide barcodes. Further error correction was performed using USEARCH version 5.2.236 [56] and UCHIME [57], and the remaining sequences were clustered into OTUs using a 97% sequence identity threshold. A representative sequence set was formed by picking the most abundant sequence from each OTU and aligned against the Greengenes core set database [58] (May 2013 version) by PyNAST [59] with a minimum sequence length of 150 and a minimum identity of 75%. The Ribosomal Database Project (RDP) classifier program [60] was used to assign taxonomy to the aligned sequences with a confidence of 0.8. The alignment was filtered prior to generating a phylogenetic tree using a lanemask to remove highly variable regions and positions that were all gaps. A phylogenetic tree was built using filtered, aligned sequences in FastTree [61] which was subsequently used to generate an unweighted UniFrac distance metric [62]. This metric included the calculated distances between samples based on OTU composition of each sample and visualized by principle coordinate analysis (PCoA).

### Functional analysis of metagenomes

Metagenome functional contents of CON, ALG, and RS diet samples were predicted using PICRUSt [22] online Galaxy version. Closed reference OTU table was generated from filtered reads (previously described) in QIIME v1.7.0 [55] using the Greengenes core set database [58] (May 2013 version) and enabling reverse strand matching. A closed reference OTU table was normalized by 16S rDNA copy number, metagenome was predicted, and they were categorized by function based on Kyoto Encyclopedia of Genes and Genomes (KEGG) pathways in PICRUSt online Galaxy version. The obtained biom file was processed by STAMP v2.0.8 [63] for statistical analysis; Welch’s *t*-test was applied to compare the KEGG pathways of diet groups pairwise (RS and CON, ALG and CON) with *P* value <0.05, confidence intervals of 95% and extended error bars were plotted. Boxplots were plotted to further focus on starch and sucrose metabolism pathway.

### Plant cell wall component (PCWCs) analysis

CoMPP was used to detect PCWCs in feed and fecal samples as described previously [64]. Two of the samples (CON.2, T6 and CON3, T5) did not contain enough material after the other analyses to be analyzed for PCWC content and were not assessed. Freeze-dried fecal samples (each of 10 mg) were homogenized by mortar and pestle. Alcohol-insoluble residues were obtained by sequential extraction using three solvents: 70% ethanol, methanol/chloroform (1:1), and acetone. Each extraction was followed by vortexing for 30 s and centrifugation at 14,500 *g* for 10 min to remove supernatant. Following this, the acetone was removed using a pipette and the samples were air dried. PCWCs were extracted from the alcohol-insoluble residues using 50 mM diamino-cyclo-hexane-tetra-acetic acid (CDTA), pH 7.5, and 4 M NaOH with 1% v/v NaBH4, which are known to solubilize pectins and noncellulosic polysaccharides, respectively. For each extraction, 300 μl of solvent was added to each tube and incubated at room temperature with shaking for 2 h. After centrifugation at 2,500 *g* for 10 min, supernatants were retained, diluted (neat, 5-, 25-, and 125-fold) in Arrayjet buffer (50% water, 50% glycerol, and 0.05% Triton X100) and the three dilutions printed in quadruplets onto nitrocellulose membranes. Every replicate was therefore represented by a 16-spot sub-array (four concentrations and four printing replicates). Arrays were probed with monoclonal antibodies (mAbs) or carbohydrate-binding modules (CBMs) (Table 1) and scanned (CanoScan 8800 F, Canon, Søborg, Denmark) and quantified using Array-Pro Analyzer 6.3 (Media Cybernetics, Rockville, MD, USA). The maximal mean spot signal was set to 100%, and all other values within that data set adjusted accordingly. A mean spot signal minimum was set as 5%.

**Table 1:** **The probes used in comprehensive microarray polymer profiling (CoMPP) and the target plant cell wall components (PCWCs)**

**Monoclonal antibody (mAb) and carbohydrate-binding module (CBM) probes**	**Target PCWCs**
LM19	Homogalacturonan (HG) partially methylesterified
INRA-RU1	Backbone of rhamnogalacturonan I
LM5	(1 → 4)-β-D-galactan
LM6	(1 → 5)-α-L-arabinan
LM21	(1 → 4)-β-D-(galacto)(gluco)mannan
BS-400-2	(1 → 3)-β-D-glucan
BS-400-3	(1 → 3)(1 → 4)-β-D-glucan
LM15	Xyloglucan (XXXG motif)
LM10	(1 → 4)-β-D-xylan
LM11	(1 → 4)-β-D-xylan/arabinoxylan
LM23	(1 → 4)-β-D-xylan
CBM3a	Cellulose (crystalline)
LM1	Extensin
JIM20	Extensin
JIM13	Arabinogalactan protein (AGP)
LM2	AGP, β-linked glucuronic acid (GlcA)
CBM20	Starch

### Statistics

The statistical significant test was applied on unweighted UniFrac distance matrices in QIIME v.1.7.0. The parametric *P* values were calculated performing two-sample *t*-tests for the pairs of the groups while nonparametric *P* values were calculated using Monte Carlo permutation (*n* = 1,000). The bacterial diversity was calculated at an OTU level using Shannon index that based on the average of ten iterations at equal subsampling size of 1,781. Analysis of covariance (ANCOVA) was run using R (version 3.1.0) package lme4 to identify the effects of time and diets on diversity of bacterial communities (based on Shannon indexes) and the relative abundances of taxa in genus and family levels. In this analysis, ALG and RS samples were compared to CON samples and the taxa with *P* value smaller than 0.01 were included in the plots. Calypso version 3.4 (http://bioinfo.qimr.edu.au/calypso/) was used to generate bubble plot to observe time-dependent changes. Each data point on bubble plot shows the mean relative abundance of bacterial families for the indicated time-point and was determined from fecal samples of three pigs that were fed with the same diet. Pearson’s correlations between the bacterial families were calculated and plotted using Calypso Version 3.4. To evaluate the interactions between gut bacteria and PCWCs over time, extended local similarity analysis (eLSA) [65,66] was performed. Cytoscape 2.7.0 [67] was used to process eLSA outputs and generate correlation networks. eLSA output was filtered by local similarity score (LS) and *P* value (*P* < 0.001) to reduce the number of nodes.

### Ethical aspects

The housing, feeding, and sampling of the animals were performed at the Nutreco Swine Research Centre facilities (Sint Anthonis, The Netherlands), and all experimental protocols describing the management, animal care, and sampling procedures were reviewed and approved by The Animal Care and Use Committee of Wageningen University (Wageningen, The Netherlands, DEC nr. 2011088.c).

### Supporting data

The sff file has been deposited in the SRA (Bioproject ID: PRJNA262976 and Accession number: SRP048624).

## Additional files

Additional file 1: Table S1. The diet ingredients and their inclusion percentages. Additional file 2: Figure S1. Rarefaction curves calculated for each diet group. Curves were calculated for observed species with standard deviation. Additional file 3: Figure S2. Shannon index variation over time. Shannon indexes were calculated to be the average of ten iterations at equal subsampling size of 1,781 for each sample. Samples were grouped by color in terms of diet group they belong to; control diet (CON) green, alginate-containing diet (ALG) blue, and resistant starch-containing diet (RS) red. Additional file 4: Table S2. Inter-individual variations and bacterial composition over time. Additional file 5: Figure S3. Bacterial family relative abundances in every sample. Different colored bars represent different families with size showing abundance of this family. Labels contain name of diet type (CON, ALG, RS), pig number for the specific diet with numbers between 1 and 3, and time point numbers between from 1 to7 in the order (starting from T1 as first time point). Additional file 6: Figure S4. Bacterial families with significantly different relative abundances between different diets. Families that have different abundances in ALG or RS pigs compared to CON pigs were determined by ANCOVA. The shown mean relative abundance percentages of the taxa were calculated using all samples taken over time within each diet. Significance degree is represented with stars; *P* < 0.05 with one star (*); *P* < 0.01 with two stars (**); *P* < 0.001 with three stars (***). The significance was stated next to the bar together with the abbreviations of compared diets (ALG, CON, and RS) when the bar does not appear for at least one of the diets due to very low relative abundance percentage. Additional file 7: Figure S5. Correlations between bacterial communities in family level. The correlations were calculated using Pearson’s correlation. Positive correlations are displayed with yellow edges and negative correlations with blue edges. The minimum similarity between the edges is 0.25. The blue nodes represent bacterial families and size of each node is proportional to the value of relative abundances. The diets (ALG, CON, RS) are shown with red nodes. Additional file 8: Figure S6. Starch and sucrose metabolism comparison of RS and CON pigs and ALG and CON pigs over time. The relative abundance of starch and sucrose metabolism pathways encoded in each imputed sample metagenome was analyzed using STAMP [54]. Time points were represented by T1 to T7 (T1: day 0, T2: day 1, T3: day 3, T4: day 7, T5: week 3, T6: week 7 and T7: week 12). Significant difference was considered only when *P* < 0.05. Additional file 9: Figure S7. Original versions of network plots in Figure 7. The networks are ordered as CON, ALG, and RS.

## Abbreviations

AGP: arabinogalactan protein
ALG: alginate-containing diet
CBM: carbohydrate-binding module
CoMPP: comprehensive microarray polymer profiling
CON: control diet
CTAB: cetyltrimethyl ammonium bromide
EDTA: ethylene diamine tetraacetic acid
eLSA: extended local similarity analysis
HG: homogalacturonan
KEGG: Kyoto Encyclopedia of Genes and Genomes
LS: local similarity score
mAb: monoclonal antibody
OTU: operational taxonomic unit
PCR: polymerase chain reaction
PCWCs: plant cell wall components
RDP: Ribosomal Database Project
RS: retrograded (type 3) resistant starch-containing diet
SDS: sodium dodecyl sulfate
SSU: small subunit
