# Supplementary figures and images for: Resistant starch diet induces change in the swine microbiome and a predominance of beneficial bacterial populations
Source: Microbiome. 2015 Apr 16;3:16. doi: 10.1186/s40168-015-0078-5 (PMC4405844; doi:10.1186/s40168-015-0078-5)

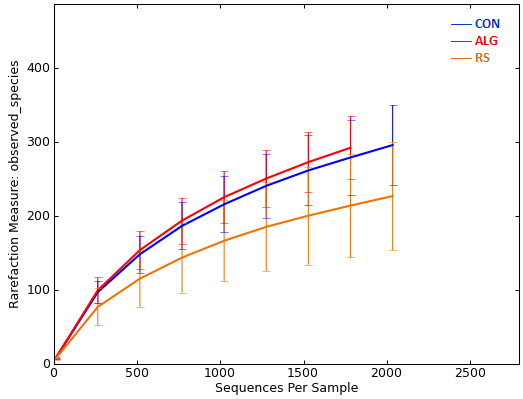

Supplement: Additional file 2: Figure S1. — Rarefaction curves calculated for each diet group. Curves were calculated for observed species with standard deviation. [file 40168_2015_78_MOESM2_ESM.tiff]

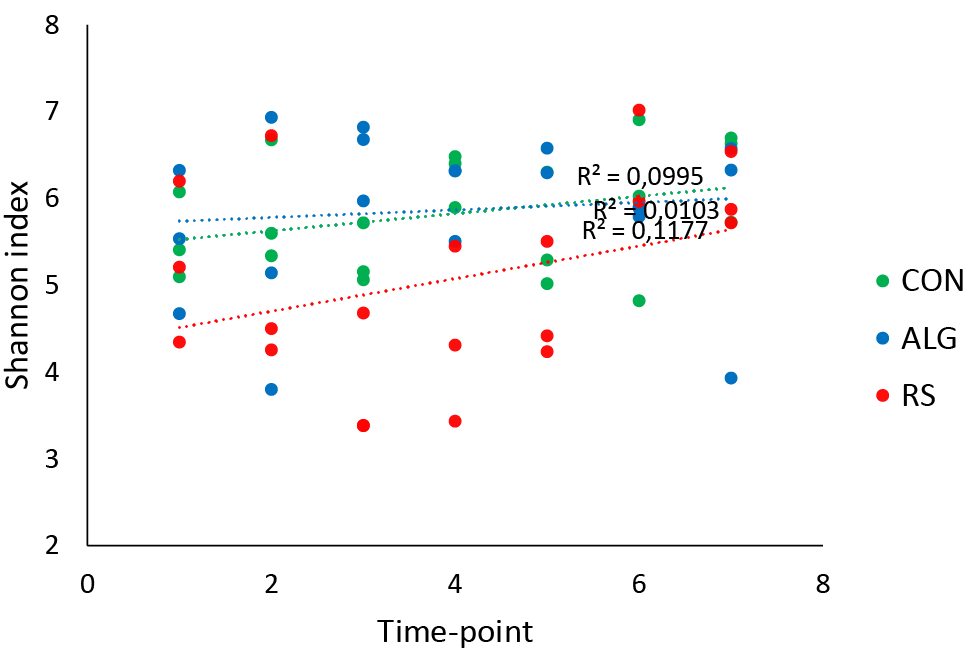

Supplement: Additional file 3: Figure S2. — Shannon index variation over time. Shannon indexes were calculated to be the average of ten iterations at equal subsampling size of 1,781 for each sample. Samples were grouped by color in terms of diet group they belong to; control diet (CON) green, alginate-containing diet (ALG) blue, and resistant starch-containing diet (RS) red. [file 40168_2015_78_MOESM3_ESM.tiff]

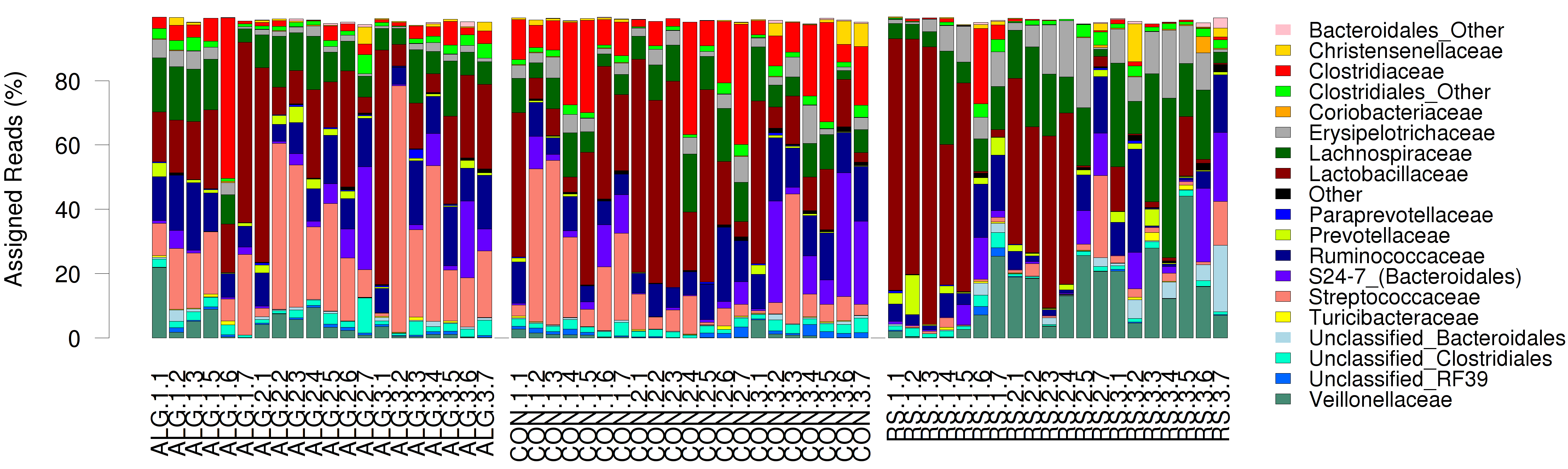

Supplement: Additional file 5: Figure S3. — Bacterial family relative abundances in every sample. Different colored bars represent different families with size showing abundance of this family. Labels contain name of diet type (CON, ALG, RS), pig number for the specific diet with numbers between 1 and 3, and time point numbers between from 1 to7 in the order (starting from T1 as first time point). [file 40168_2015_78_MOESM5_ESM.tiff]

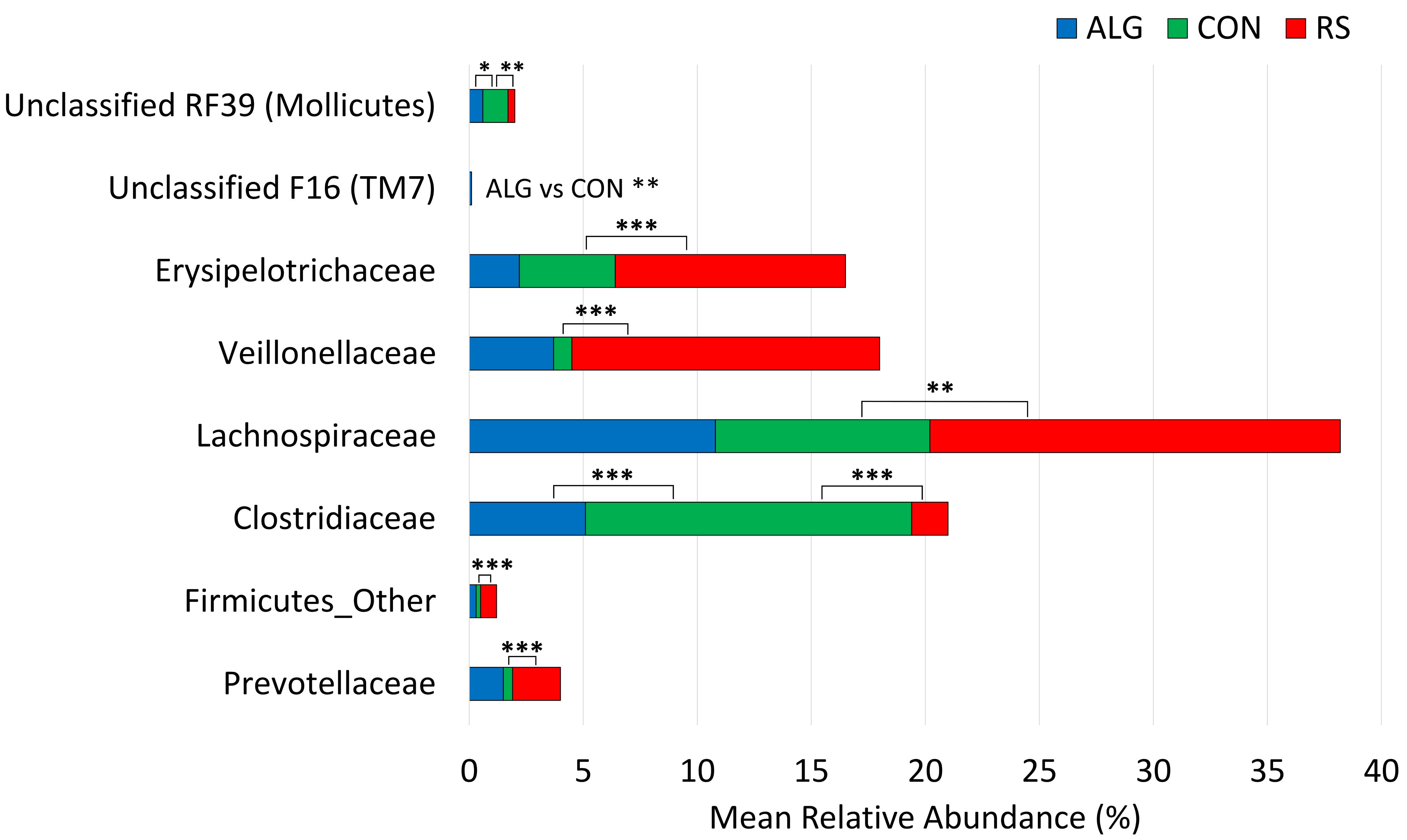

Supplement: Additional file 6: Figure S4. — Bacterial families with significantly different relative abundances between different diets. Families that have different abundances in ALG or RS pigs compared to CON pigs were determined by ANCOVA. The shown mean relative abundance percentages of the taxa were calculated using all samples taken over time within each diet. Significance degree is represented with stars; P < 0.05 with one star (*); P < 0.01 with two stars (**); P < 0.001 with three stars (***). The significance was stated next to the bar together with the abbreviations of compared diets (ALG, CON, and RS) when the bar does not appear for at least one of the diets due to very low relative abundance percentage. [file 40168_2015_78_MOESM6_ESM.tiff]

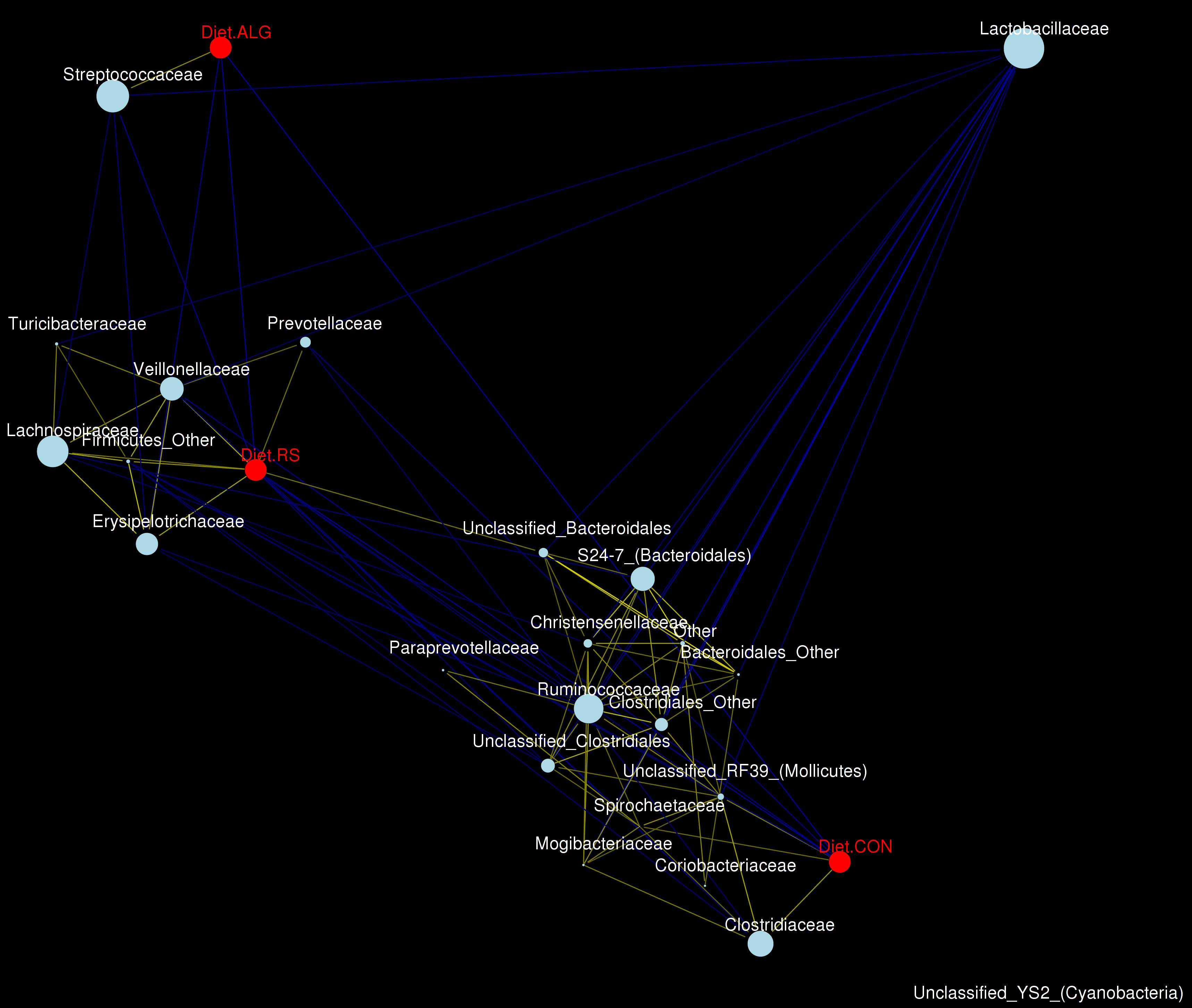

Supplement: Additional file 7: Figure S5. — Correlations between bacterial communities in family level. The correlations were calculated using Pearson’s correlation. Positive correlations are displayed with yellow edges and negative correlations with blue edges. The minimum similarity between the edges is 0.25. The blue nodes represent bacterial families and size of each node is proportional to the value of relative abundances. The diets (ALG, CON, RS) are shown with red nodes. [file 40168_2015_78_MOESM7_ESM.tiff]

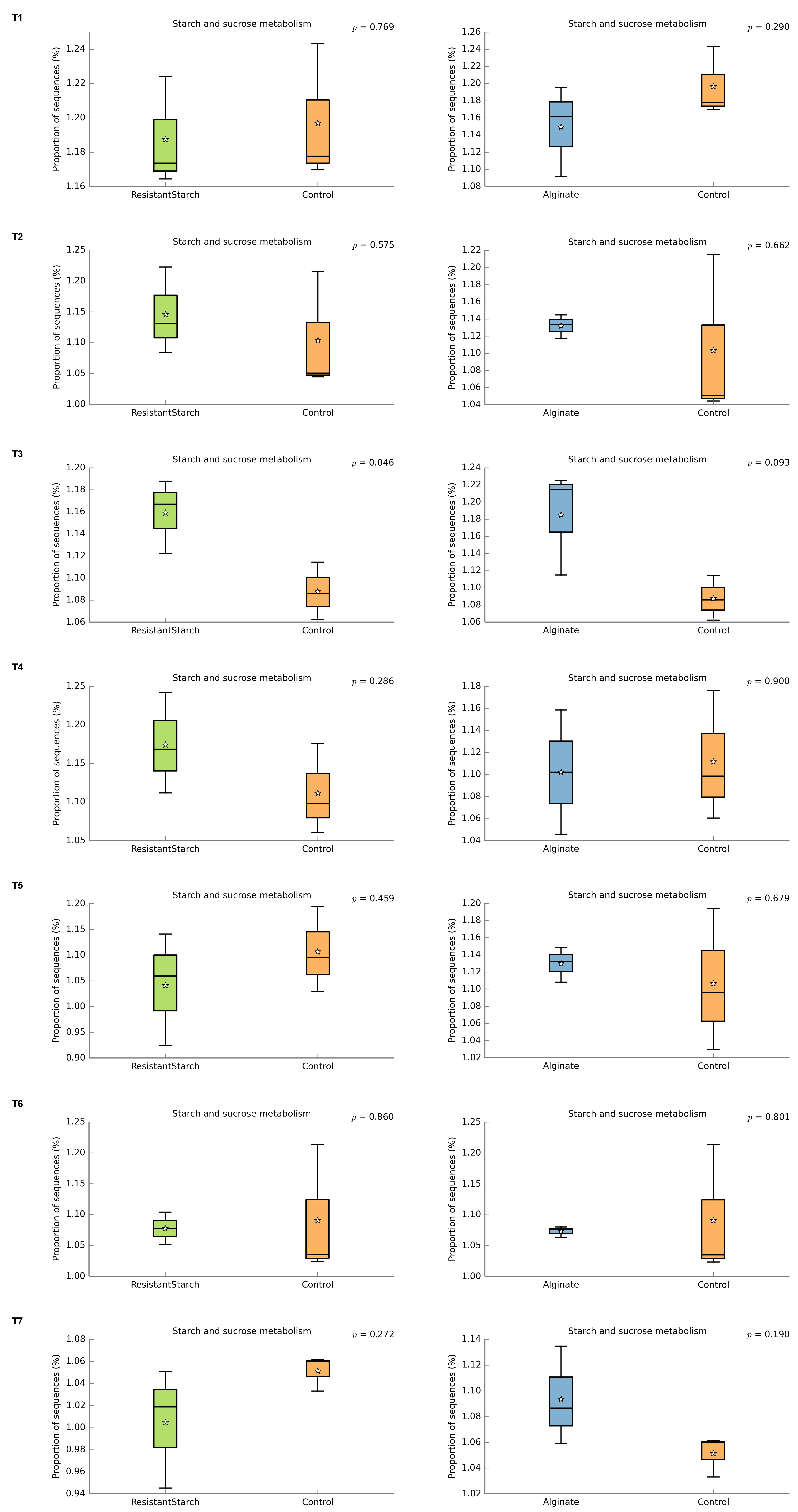

Supplement: Additional file 8: Figure S6. — Starch and sucrose metabolism comparison of RS and CON pigs and ALG and CON pigs over time. The relative abundance of starch and sucrose metabolism pathways encoded in each imputed sample metagenome was analyzed using STAMP [54]. Time points were represented by T1 to T7 (T1: day 0, T2: day 1, T3: day 3, T4: day 7, T5: week 3, T6: week 7 and T7: week 12). Significant difference was considered only when P < 0.05. [file 40168_2015_78_MOESM8_ESM.tiff]

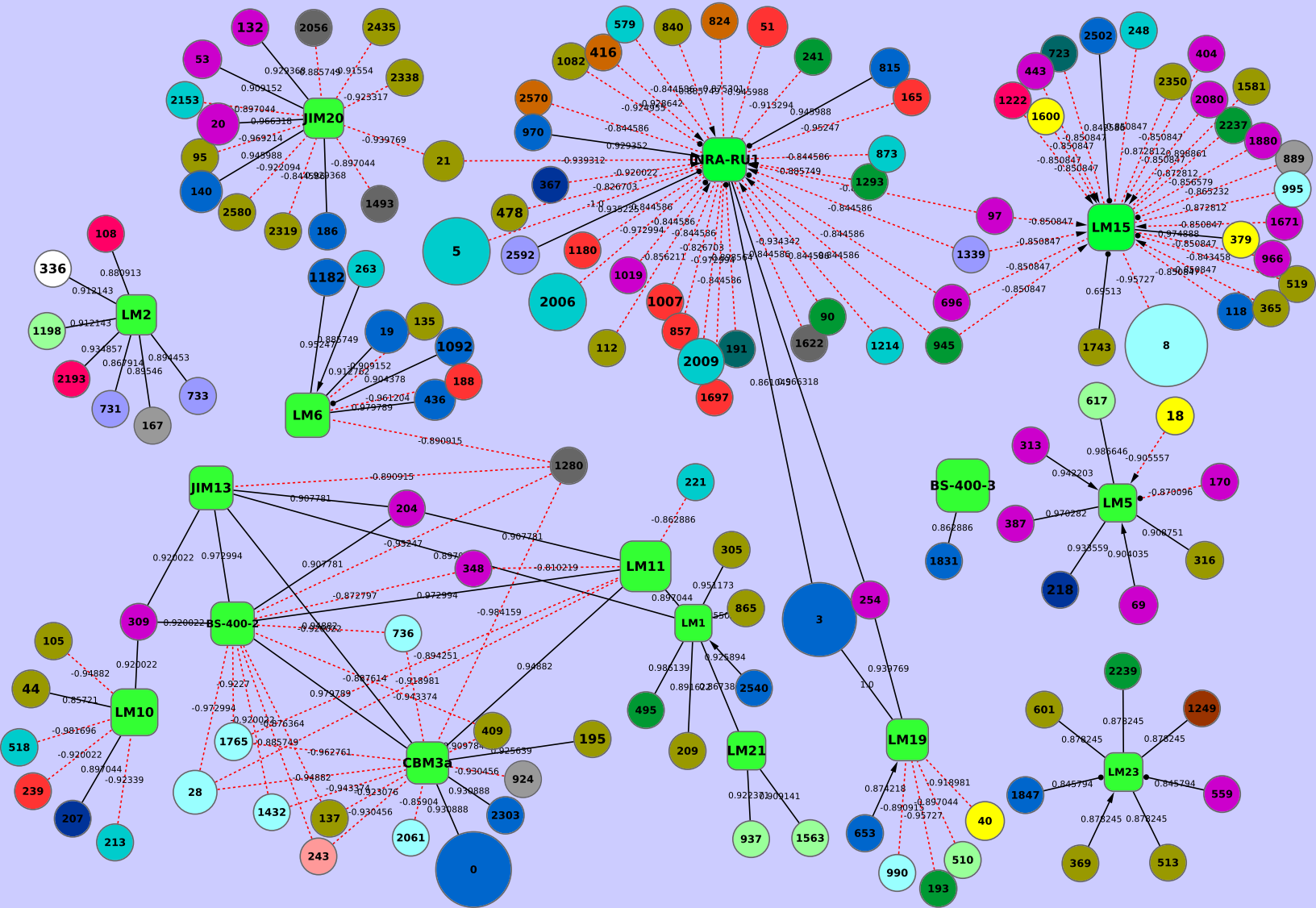

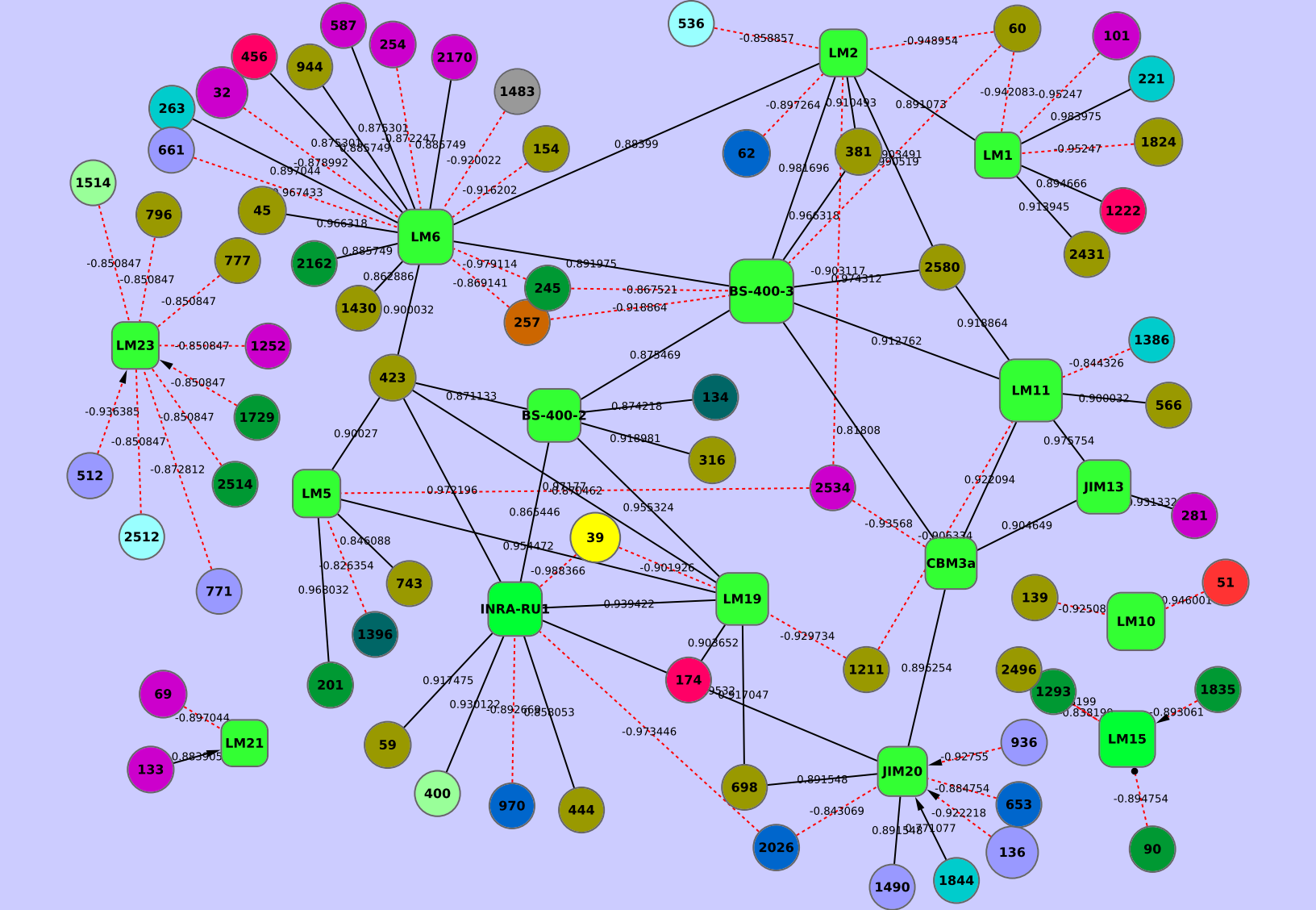

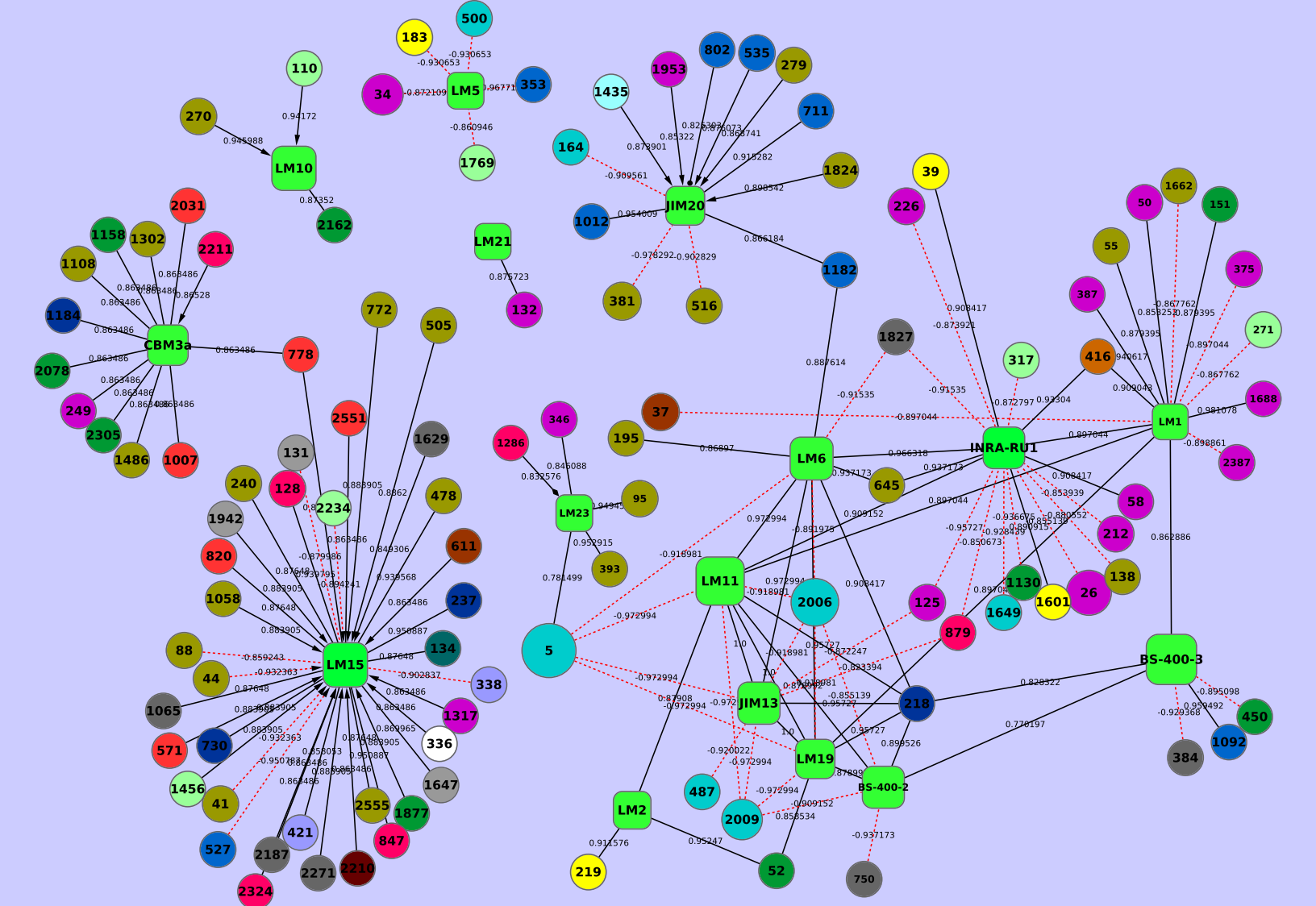

Supplement: Additional file 9: Figure S7. — Original versions of network plots in Figure 7. The networks are ordered as CON, ALG, and RS. [file 40168_2015_78_MOESM9_ESM.pdf]
